# Dysfunction of the Blood-Brain Barrier—A Key Step in Neurodegeneration and Dementia

**DOI:** 10.3389/fnagi.2020.00185

**Published:** 2020-07-24

**Authors:** Christian R. Noe, Marion Noe-Letschnig, Patricia Handschuh, Chiara Anna Noe, Rupert Lanzenberger

**Affiliations:** ^1^Department of Medicinal Chemistry, University of Vienna, Vienna, Austria; ^2^ProFem GmbH, Vienna, Austria; ^3^Neuroimaging Lab (NIL), Department of Psychiatry and Psychotherapy, Medical University of Vienna, Vienna, Austria; ^4^Department of Otorhinolaryngology, University Clinic St. Poelten, St. Poelten, Austria

**Keywords:** blood-brain barrier (BBB), GLUT1, dementia, Alzheimer’s disease, amyloid precursor protein (APP), inflammation, positron emission tomography (PET), glucose

## Abstract

The vascular endothelium in the brain is an essential part of the blood-brain-barrier (BBB) because of its very tight structure to secure a functional and molecular separation of the brain from the rest of the body and to protect neurons from pathogens and toxins. Impaired transport of metabolites across the BBB due to its increasing dysfunction affects brain health and cognitive functioning, thus providing a starting point of neurodegenerative diseases. The term “cerebral metabolic syndrome” is proposed to highlight the importance of lifestyle factors in neurodegeneration and to describe the impact of increasing BBB dysfunction on neurodegeneration and dementia, especially in elderly patients. If untreated, the cerebral metabolic syndrome may evolve into dementia. Due to the high energy demand of the brain, impaired glucose transport across the BBB *via* glucose transporters as GLUT1 renders the brain increasingly susceptible to neurodegeneration. Apoptotic processes are further supported by the lack of essential metabolites of the phosphocholine synthesis. In Alzheimer’s disease (AD), inflammatory and infectious processes at the BBB increase the dysfunction and might be pace-making events. At this point, the potentially highly relevant role of the thrombocytic amyloid precursor protein (APP) in endothelial inflammation of the BBB is discussed. Chronic inflammatory processes of the BBB transmitted to an increasing number of brain areas might cause a lasting build-up of spreading, pore-forming β-amyloid fragments explaining the dramatic progression of the disease. In the view of the essential requirement of an early diagnosis to investigate and implement causal therapeutic strategies against dementia, brain imaging methods are of great importance. Therefore, status and opportunities in the field of diagnostic imaging of the living human brain will be portrayed, comprising diverse techniques such as positron emissions tomography (PET) and functional magnetic resonance imaging (fMRI) to uncover the patterns of atrophy, protein deposits, hypometabolism, and molecular as well as functional alterations in AD.

## Introduction

The human brain’s activity is driven by about 86 billion neurons and 85 billion glial cells, mostly astrocytes (Azevedo et al., 2009) that are connected by a highly complex axonal network. Once embedded and stabilized in this network, neurons are maintained for a lifetime. The points of axonal contact are the synapses, where neuronal signals are transmitted by neurotransmitters from neuron to neuron, molecule by molecule (Jessell and Kandel, 1993). Glia cells of the brain do not only support and isolate neurons but are of utmost importance for metabolic functions. The maintenance of the continuous activity of this communication system by synthesis, release, and reuptake of neurotransmitters is a tremendous energetic effort for both neurons and glial cells (Habbas et al., 2015; Santello et al., 2019). The decline in metabolic activity during aging also includes a reduced capacity for the synthesis of neurotransmitters, which means that the general decline in cognitive activity in elderly people is to be considered a “natural” process. Consequently, physical and mental decline is directly related to the exhaustion of synaptic vesicles due to a lack of metabolites (Ivakhnitskaia et al., 2018).

Beyond that, neuronal cells are endowed with synaptic plasticity, which allows the strengthening of pre-existing axonal connections and the formation of new ones as a reaction to new, repetitive, or intense impulses (Draganski et al., 2004; Bruel-Jungerman et al., 2007). Stem cell transformation into neurons has recently become a further element of this system (Selvaraj et al., 2012). Neuronal plasticity equally requires an efficient and continuous supply with nutrients and metabolites. The reduced synthetic capacity for metabolites going along with aging is not limited to neurotransmitters, but also affects the components (e.g., phospholipids) of the neuronal cell membrane. Phospholipids are needed to strengthen neuronal connections within the process of synaptic plasticity. Therefore, also synaptic plasticity is weakening with age (Power and Schlaggar, 2017). Reduced neuronal plasticity can either be of transient or permanent character, depending on whether new impulses are set by enduring activity or not. Mental activity means “training impulses” for neurons to preserve neuronal stability and to induce neuronal growth *via* synaptic stability (Power and Schlaggar, 2017). If certain pre-existent neuronal pathways are not used anymore, the inter-synaptic communication pathway can get lost, resulting in irreversible memory loss. This explains why both mental and physical activity is of utmost importance for aging people (Draganski et al., 2004). However, it must be borne in mind that this “normal” impairment or loss of “unused” inter-neuronal connections does not correspond to the massive decline in inter-axonal communication processes going along with neuronal cell death, as seen in the course of dementia, in particular in Alzheimer’s disease (AD; Guillon et al., 2017; Power and Schlaggar, 2017).

The term dementia describes a pathology or a syndrome that is characterized by an increasing loss of cognitive, social, and emotional abilities (Cunningham et al., 2015). This process is associated with an abnormal level of cellular decline within the brain that exceeds regular aging (Simić et al., 1997). Since dementia is usually linked to higher age, the relevance of mental disorders is constantly growing. At present, cardiovascular disease (in the narrower sense, cardiac failure, and stroke) is the leading cause of death worldwide (Roth et al., 2017). Nevertheless, dementia as a cause of death has been gaining importance progressively in recent years. Among other causes, this can be traced back to demographic change with a higher average life expectancy in industrial as well as developing countries. A wide range of manifestations of neurodegeneration has been defined, with AD representing the most frequent one (60%; Alzheimer’s Association, 2017). Between 2000 and 2016, the mortality caused by AD has doubled, reaching the position of the fifth-leading cause of death (World Health Organization, 2018).

A variety of mechanisms can cause neurodegeneration. Overlaps between diverse forms of dementia may further complicate diagnostics (Iadecola, 2010). In some cases, responsible triggers have been identified. Nevertheless, there are significant gaps that need to be closed regarding the etiology of neurodegeneration, especially in the case of AD. Regarding their pathomechanism, an increasing number of commonalities have been identified across the diverse forms of dementia and cardiovascular diseases (Ashraf et al., 2016). Such a commonality also appears with diabetes mellitus, which is not only associated with vascular comorbidities but is also known to increase the risk of AD (Arvanitakis et al., 2004; Pilon, 2016). Several forms of dementia are associated with pathological vascular alterations (O’Brien and Thomas, 2015), thus linking neurodegeneration and cardiovascular disease. In this context, the role of the blood-brain barrier (BBB) in neurodegeneration has recently gained growing attention (Weiss et al., 2009; Blonz, 2017; Yamazaki and Kanekiyo, 2017) and will be the main focus of this article.

## The Cerebral Metabolic Syndrome—A Coherent Concept for Preventing and Treating Neurodegeneration

### The Blood-Brain Barrier

Atherosclerosis is one of the well-known results of dysfunctional endothelial tissue within the vessel walls. The pathophysiological consequences of unfavorable lifestyle habits (e.g., overeating, smoking, lack of exercise) and other, endogenous factors (e.g., genetic vulnerability) are summarized under the term “metabolic syndrome” (MS), which describes the cluster of obesity, elevated blood lipid levels, hypertension and impaired glucose tolerance (Grundy et al., 2004). It represents the common pathologic starting point for secondary diseases like diabetes mellitus, myocardial infarction, and stroke. Its growing global prevalence comes along with the continuous increase of obesity due to the mentioned unfavorable living conditions and eating habits. Summing up these conditions into one clinical picture has proven its worth in respect to prevention and therapy (Mendrick et al., 2018).

The brain as the central controlling structure of the body, its strict separation from the remaining physiological systems—especially the blood system—is reasonable. This separation is ensured by a specific structure of the vascular endothelium in the brain. Induced by factors released by brain astrocytes, vascular endothelial cells express intercellular adhesion molecules (most importantly, claudin and occludin) that connect them *via* tight junctions (Neuhaus et al., 2008; Weiss et al., 2009). The resulting tight cerebral vascular endothelium and additional components are building up the BBB, separating brain tissue from the blood. This tight barrier exhibits a high electric resistance and does not allow peri-cellular transport (Neuhaus et al., 2012; Novakova et al., 2014) into the brain. Apart from passive diffusion of lipophilic molecules, any transport into and out of the brain’s nervous tissue is exclusively transporter-mediated (Kniesel and Wolburg, 2000; Wolburg and Lippoldt, 2002; Ecker and Noe, 2004; Shah et al., 2012). Bearing this in mind, it is evident that the impairment of membranous fluidity and functionality going along with atherosclerosis might harm brain cells even more than peripheral ones. Insufficient supply of nutrients and metabolites into the brain over the BBB is the primary cause for a process, which might be called cerebral metabolic syndrome. This finding is crucial for and consistent with increasingly upcoming hypotheses, relating dementia to metabolic dysfunction or energy deficits of the brain (Jurcovicova, 2014; Szablewski, 2017). In contrast to atherosclerosis and the related metabolic syndrome, the fundamental consequences of BBB impairment have only recently become the focus of broader attention (Noe, 2009; Yamazaki and Kanekiyo, 2017). A synoptic mechanistic view towards metabolic syndrome and cerebral metabolic syndrome to define potential commonalities is still missing, although they might appear as the first signs of pathophysiological processes, leading to neurodegeneration as a potentially life-threatening syndrome.

### The Energy Supply of the Brain

**Figure 1:**
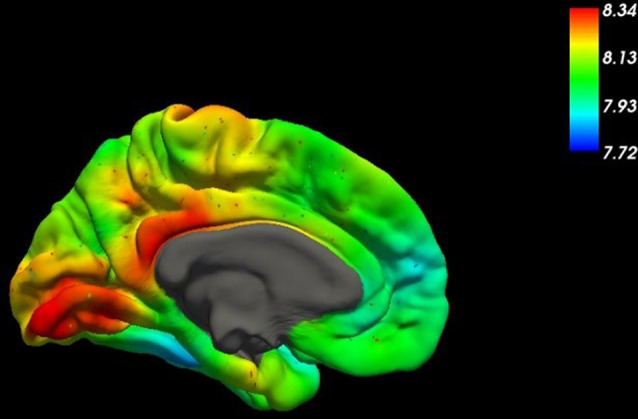
This figure shows the spatial distribution of the mRNA expression of SLC2A1, the gene coding for the glucose transporter GLUT1, across the medial surface of the human brain (Gryglewski et al., 2018). Higher concentrations are indicated by red colors in the table.

The plethora of physiological brain activities explains the high energy demand of the brain. Not surprisingly, the brain is by far the largest consumer of glucose, physiologically the main energy source of most organisms. About 200 g of glucose are needed per day just to sustain the brain’s basal metabolism (Peters, 2011). This amount corresponds to about 25–50% (Reinmuth et al., 1965) of available (mobilizable) glucose within the organism (Fehm et al., 2006). Due to the steadily high demand for glucose, it is evolutionarily implicated that the glucose-transport into the brain—to a large extent—does not depend on the insulin system (Thorens and Mueckler, 2010), which is a signaling system that is directing blood glucose into tissues upon demand. As a result of insulin signaling, cells express glucose transporters (GLUT4), which then transport glucose into the cell. In contrast to these, GLUT1 (Figure 1) glucose transporters at the BBB are independent of insulin signaling. They secure the continuous transport of glucose across the BBB (Shah et al., 2012).

There is convincing evidence that also the efficiency of GLUT1 transporters might be affected by a loss of membrane fluidity (Pifferi et al., 2005; Deng et al., 2015; Winkler et al., 2015). It might even be speculated that the up-regulation of blood glucose levels could be triggered by an impairment of BBB transport. Nevertheless, it is broadly assumed that the adequate energy supply of the brain is warranted in any case (Fehm et al., 2006). Thereby, various pathways can be activated, including the utilization of lactate and ketone bodies in case of acute hypoglycemia, for instance (Morris, 2005; van Hall et al., 2009).

The mentioned loss of membrane fluidity is caused by various pathophysiological mechanisms, all harming the transport function and stability of the BBB. Therefore, cerebral glucose availability in obese people tends to be impaired despite chronic hyperbolic nutrition (Wardzinski et al., 2018). High blood sugar levels in diabetic patients do not guarantee an adequate supply of glucose to the brain. Life-threatening situations, which may occur in cases of severe hypoglycemia, and which are a frequent and well-known danger in diabetic patients, point to the possibility that the maintenance of a physiological level of glucose in the brain is by no means self-fulfilling in any case.

Inadequate glucose availability in the brain is an important element of impaired function going along with cerebral metabolic syndrome. However, the consequences go far beyond mere impairment. Apoptosis—the controlled dismantlement of a cell from the inside—is usually initiated within mitochondria, which control the energetic metabolism of a cell (Hengartner, 2000; Martinvalet et al., 2005). With glucose being the main energy source of the brain, one may assume that a permanent and adequate availability of glucose in the brain is crucial to delay mitochondrial apoptotic processes. Lack of glucose has been directly associated with neuronal cell death (Mergenthaler et al., 2013). A permanent deficit in glucose availability renders a brain more vulnerable to neurodegenerative processes.

### Definition

We propose to use the term “cerebral metabolic syndrome,” by widening the definition of the “metabolic syndrome.” Both syndromes are related to endothelial dysfunction of blood vessels, usually starting from a loss of endothelial membranous fluidity. In the case of cerebral metabolic syndrome, this dysfunction is focused on the BBB. In its first manifestation, it describes a clinical situation by which a reduced neuronal capacity evolves into symptoms such as mental fatigue, obliviousness, stress aversion, or depression. However, focused on BBB pathophysiology, the concept goes far beyond a mere description of aging deficits. Impairment, dysfunction, and destruction of the BBB system is not the only causative for the clinical picture of improper aging, but also onset and/or aggravation of dementia.

The cerebral metabolic syndrome may also go along with hypertension and impaired glucose tolerance, which are the main consequences of metabolic syndrome. However, the syndromes will lead to different secondary diseases, unless treated timely. In the case of metabolic syndrome, hypertension is related to myocardial infarction and/or stroke (Isomaa et al., 2001), while impaired glucose tolerance is the prodromal stage of diabetes (Mendrick et al., 2018). In the case of the cerebral metabolic syndrome, however, hypertension and glucose intolerance additionally point to a potentially impaired transport function of nutrients and metabolites into the brain across the BBB. Increased blood pressure and elevated blood sugar levels are also indicators signaling impending neurodegeneration and dementia.

Over the last two decades, there has been a shift, from reductionist towards systemic approaches, favoring synoptic views. According to this, all specific types of dementia may be considered as part of one group of brain diseases and may be expected to have certain features in common. Further, the systemic approach implies that brain functions should be interpreted concerning the rest of the body, considering them as part of an interdependent system. The BBB, as a bidirectional interface between the central nervous system and the periphery, serves as an integral element in this context. The cerebral metabolic syndrome is combining a certain number of clinical pictures into one conceptual system, thus providing a frame for systemic approaches in understanding and treatment of neurodegeneration. Defining the clinical picture of the metabolic syndrome has been particularly helpful because it connects individual lifestyles to severe, life-threatening diseases. In times of genetic determinism, the concept of metabolic syndrome helped to point out the individual responsibility for personal health. Thereby, the main focus on preventing obesity is needed, which is a primary factor for metabolic syndrome and a health problem increasingly affecting young people (Dominguez and Barbagallo, 2016). In the case of the cerebral metabolic syndrome, aggravation of metabolic deficits in elderly people by impaired function of their BBB is the major cause of disease. The opportunity of linking lifestyle to life-threatening diseases might trigger the feeling of responsibility for personal health, also on the field of neurodegeneration. This is one of the major justifications for defining cerebral metabolic syndrome.

### Diagnosis of Cerebral Metabolic Syndrome

Considering the overlap of both syndromes, most diagnostic parameters in use for the metabolic syndrome are applicable for the cerebral metabolic too (Grundy et al., 2005). Despite these methods, various diagnostic tools exist, which allow for determining mental performance, cognitive deficits, and other symptoms. Mental testing is in the focus of cerebral metabolic syndrome diagnostics.

The lost neuronal function can hardly be replaced (Power and Schlaggar, 2017). Therefore, therapy of neurodegeneration is to start as soon as first clear signals come up or before. Most of the diagnostic methods in use to detect loss of mental functions are of empirical character (Fleming et al., 1995), however, brain imaging methods (that will be discussed later on) have gained widespread attention over the last decades. A standardized set of diagnostic methods still is to be set up for the cerebral metabolic syndrome. This set might include the screening for biomarkers, predicting the risk of developing dementia later on. The diagnostic tools will have to include the possibility to examine metabolic functions, such as the brain’s energy supply in general, and the impairment of BBB functioning, specifically. Existing metabolic parameters, such as homocysteine (Derosa et al., 2006) and other metabolites, as well as enzymes and coenzymes, should be screened and adjusted to serve as predictors for pathologic processes. Given the rapidly evolving field of imaging methods, further development in this area will hopefully lead to sufficient prediction accuracy in diagnosing the cerebral metabolic and sequelae with high specificity and sensitivity (Aisen et al., 2017).

### Prevention

Given its life-threatening sequelae, the prevention of metabolic syndromes is a central goal, both in the peripheral and the cerebral case. With both syndromes, dietetic measures, as well as physical exercise, are particularly important. Measures include diets to maintain vascular membrane fluidity. Increased use of fats with a high content of specific unsaturated fatty acids, e.g., omega-3 fatty acids, avoidance of trans-fats, and cholesterol-rich food are known to support membrane fluidity both in younger and older people (Gammone et al., 2018). In the classification of the individual nutritional status, the definition of the body mass index (BMI) has proven to be a helpful marker to control dietary measures and to foster health competency within the population (Afshordel et al., 2015; Cass, 2017). With cerebral metabolic syndrome, the improvement of physical fitness should be complemented by measures to maintain mental fitness. Cognitive training to promote and preserve neuronal plasticity is of special interest, also to prevent a cerebral metabolic syndrome in elderly people (Meeusen, 2014).

In the prevention of cerebral metabolic syndrome, specific attention should be attributed to brain nutrition, most of all, to the energy supply of the brain by providing sufficient glucose. While in most tissues, energy can also be provided by other sources, mainly other carbohydrates such as fructose, mannose, and lactose, the main consumers of glucose—brain tissue and erythrocytes—entirely dependent on the supply of molecular glucose (Mergenthaler et al., 2013). Due to the lack of a nucleus, erythrocytes cannot express enzymes to utilize other sources than glucose. The brain, on the other hand, depends on the active transport of glucose over the BBB, because its relatively high energy demand cannot be covered by alternative metabolic pathways on the long-term impact (Hajjawi, 2013).

## Dementia

### From Cerebral Metabolic Syndrome to Dementia

The risk of developing a specific type of dementia depends on genetic as well as epigenetic factors, including lifestyle and nutrition habits. By now, several genes could be identified that are known to be involved in AD, but none of them is exclusively accountable for this type of dementia (Broadstock et al., 2014). The disease rather originates from a combination of a dysfunctional genomic network, including impaired alleles of receptors, transporters or enzymes, and specific lifestyle parameters. Extensive ongoing research on neurodegeneration has generated a wealth of new knowledge and will certainly add new targets and novel drugs for therapy. Due to its strict obligation for active transport, the BBB provides—beyond GLUT1—a series of potentially dysfunctional receptors and transporters. They are determinants for the course of neurodegeneration (Baumgart et al., 2015) and might serve as drug targets.

Nevertheless, in our opinion, it cannot be expected that a reductionist concept leading to one perfect drug-target will appear to provide the “total” solution for AD. Indeed, the evaluation of holistic concepts seems to be a promising approach. The cerebral metabolic syndrome, particularly the impaired function of the BBB, is proposed as the starting point of this path, where dementia could be interpreted as a consequence of cerebral metabolic syndrome (Iadecola, 2010). Thereby, neurodegeneration might be induced by an overall impairment of BBB functioning, including loss of fluidity as well as (partly genetically determined) deficits of receptors and transporters in the BBB. Inflammation (Heneka et al., 2015) or infections (Holmes et al., 2003) of BBB tissue could aggravate vascular dementia (VD) and induce AD. Not only the endothelial transport capacity will be disturbed in the course of such processes, but also the integrity of the BBB is threatened by apoptotic procedures, thus carrying neurodegeneration into deeper compartments of the brain.

### The Choline System and Apoptosis

Cellular survival is largely modulated by apoptosis and autophagy, which has gained increased attention in anti-aging and neurodegenerative research recently (Gump and Thorburn, 2011). In the human body, millions of new cells arise every day. At the same time, millions of them decay. This type of cellular death is an efficient, controlled process called apoptosis. It guarantees the disposal of dying cells without disturbing ongoing, physiologic processes within the organism (Kerr et al., 1972; Elmore, 2007). Brain cells, especially neurons, are highly durable and cannot be simply reproduced. They are part of a highly complex network with an individual set of connections to other neurons. Neuronal cell loss is, therefore, mostly irreversible damage (Okouchi et al., 2007). The status of cerebral metabolic syndrome supports apoptotic processes and cellular decay due to inflammation.

It has long been known that the pathogenesis of AD comes along with a deficiency in acetylcholine. Therefore, the therapy of AD includes the administration of acetylcholinesterase inhibitors (Ladner and Lee, 1998), substances that are meant to prevent the breakdown of acetylcholine. It is known that these drugs improve the current state of concerned patients, though they lack an effect on disease progression (Bartolini et al., 2003). Acetylcholine is formed by acetylation of choline. It is well known that choline is also a structural moiety of phosphatidylcholine, which is the major component of the lipid bilayer of cell membranes. A lack of choline can mean both a lack of acetylcholine—the deficient neurotransmitter in AD—and a lack of the membrane constituent phosphatidylcholine (Hollenbeck, 2012).

In the course of apoptotic dismantling, the cell shrinks, splitting cellular DNA into typical fragments. At the end of this internal process, parts of the inner layer of the cell membrane that are rich in the phospholipid phosphatidylserine change their position. They flip to the outer surface of the apoptotic cell, replacing phosphatidylcholine rich epitopes. Now, instead of choline, the amino acid serine is the polar rest of the outer cell membrane (Fadok et al., 1992; Ravichandran, 2011). These phosphatidylserine epitopes at the outside are the signals tagging the cell as “disposable.” Thereby, a well-known cascade of final steps of apoptotic cell death is induced, attracting macrophages to internalize the tagged apoptotic cell (Ravichandran, 2011).

The cross-relation of both acetylcholine and phosphatidylcholine deficiency has not been considered sufficiently up to now. We hypothesize that the choline-related impairment of cell metabolism, which results in a cellular acetylcholine-deficiency, will at the same time have an impact on the composition of the cell membrane, increasing there the relative share of the phosphatidylcholine-precursors phosphatidylserine and phosphatidylethanolamine. While the acetylcholine-deficiency can be held accountable for emerging neurological deficits of AD, the lack of phosphatidylcholine—due to the same metabolic defect—impairs neuronal plasticity. Above all, it directly triggers the induction of the apoptotic cascade. This might explain why the administration of acetylcholinesterase inhibitors or acetylcholine receptor agonists improves physiologic deficits only temporarily (Rogers et al., 2000; Ferreira-Vieira et al., 2016).

In terms of chemical reactions, the biosynthesis of choline (Bremer et al., 1960) starts with the decarboxylation of the amino acid serine to ethanolamine, followed by the quaternization of ethanolamine, which is affected by methylation with three methyl groups. Thereby, the choline molecule receives its typical pH-independent polar charge. This sequence of steps is relevant both in acetylcholine- and phosphatidylcholine-biosynthesis (Hollenbeck, 2012). The provision of methyl- and methylene-groups (one carbon building blocks) is the task of a complex metabolic system, the choline system. Among others, it comprises the coenzymes folic acid (B9) and cobalamin (B12), as well as the amino acid methionine, which are substrates of closely interacting enzymes. In this context, S-adenosylmethionine, formed from methionine (Mato et al., 1997), plays a major role. Adenosylation activates the methyl group of methionine for methylation. In the course of this process, homocysteine is generated from methionine, a marker for both the ongoing active methylation and for the dysfunction of the methylation cycle. The dysfunction of the choline system may cause severe deficits. It plays an important role both in embryonal development and in the process of aging. It may be speculated that an insufficient biosynthesis of choline directly triggers apoptotic cellular loss in all cells of the body, a phenomenon that is known to be accelerated in patients with AD (Campoy et al., 2016; Bekdash, 2018).

Cellular availability of the components of the choline system depends on their active transport across the vascular endothelium. Any loss of membrane fluidity will, therefore, harm the system. However, the situation is by far aggravated in the brain, where the tight BBB will potentiate the effects of endothelial transport deficits. This is also true for the amino acids and coenzymes of the choline system (Fadok et al., 1992; Hollenbeck, 2012; Bekdash, 2018). We hypothesize that a dysfunctional blood-brain might trigger apoptotic processes also by preventing adequate availability of methyl groups, required both for the biosynthesis of acetylcholine and phosphatidylcholine. In any case, both impaired glucose transport—inducing mitochondrial apoptosis—and an impaired choline system—speeding up apoptotic processes by macrophage attraction—are directly related to cellular apoptosis and will render neuronal cells more susceptible to neurodegenerative processes in general (Okouchi et al., 2007). They are candidates for a rational interpretation of neuronal cell death in normal aging processes and VD.

### Vascular Dementia

A steadily decreasing function of BBB endothelial cells may support the pathological process of a slowly proceeding dementia. However, increasing vascular deficits may equally end up in a local mechanic deconstruction of a brain vessel leading to VD. There, ischemic or hemorrhagic infarcts may affect multiple brain areas (Iadecola, 2010) due to occlusion of vessels or due to the loss of microvascular tightness. The situation is different with AD, which is diagnosed per definition by the appearance of amyloid plaques.

## A Hypothesis on the Etiology of Alzheimer’s Disease

### The Amyloid Precursor Protein (APP)

Although a wide range of approved medicines is available for palliative therapy of neurodegeneration, the present pharmacotherapeutic situation is far away from satisfactory given the rising prevalence of dementia (Ladner and Lee, 1998; Ferreira-Vieira et al., 2016). For more than 30 years, AD has been one of the central areas of academic and industrial research. However, no significant breakthrough regarding therapeutic approaches has been achieved so far (Salloway et al., 2008). The most characteristic pathologic features of AD are the amyloid plaques found in the brain (Gouras et al., 2010). It is almost self-evident to assume that exactly these plaques impair the functionality of cells and cause cell death in the long-term. Up to now, all efforts to develop drugs that aim to dissolve these plaques and remove them from the brain have not yet been successful. This also holds true for those immunotherapies that target APP (Sigurdsson et al., 2001; Solomon and Frenkel, 2010) and Tau, the second pathophysiological protein in AD. Currently, a disappointment on the slow progress is visible in industrial research, which has led to the cancellation of research activities performed by major pharmaceutical companies in this field (Broadstock et al., 2014). While the diagnosis of neurodegeneration is well established, a reasonable approach to counteract neurodegeneration, above all AD, is still lacking.

Genetic investigations have revealed significant APP mutations causally linked to a familial predisposition for AD (Cuyvers and Sleegers, 2016). However, an exclusive role of genetic factors could only be shown for a low percentage of all AD patients—a subset of less than 10% so far (Zhang et al., 2011).

**Figure 2:**
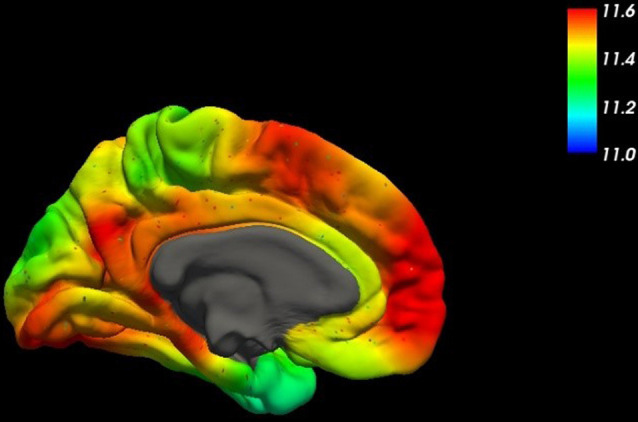
This figure shows the spatial distribution of the mRNA expression of the amyloid-β precursor protein (APP) across the human brain (mesial surface) using high-resolution mapping techniques of the human whole-brain transcriptome (Gryglewski et al., 2018).

For all the other cases, a rather complex etiology is to be assumed with amyloid plaques as a common marker. Even though amyloid plaques are probably rather remnants of a devastating pathophysiologic process than elicitors, the central role of the precursor protein APP (Figure 2) in AD remains unquestioned (Goate et al., 1991).

### Inflammation of BBB Cells

Already in 1989 (Braak et al., 1989), a detailed study on the presence of cerebral amyloid tangles in deceased patients appeared (Braak and Braak, 1991). These investigations were also beneficial concerning understanding the etiology of AD. Two remarkable correlations have emerged from this research. First, the plaques could be found scattered across the whole study population; they were not limited to patients suffering from AD. Second, an age-dependent increase in plaque formation could be identified. These findings shed doubt on the assumption that the amyloid plaques are the primary mandatory cause of dementia. Probably these results were relevant for upcoming concepts, which relate AD to inflammatory processes of the brain (Neuhaus et al., 2011; Hommet et al., 2014; Blonz, 2017). Although treatment of inflammation seems to be a manageable task, the situation is different and by far more complex with chronic inflammation of unproven origin (Dominy et al., 2019). Although sporadic success has been shown for the inflammation concept over the years, it has remained a therapeutic outsider approach, also because a lifelong preventive drug therapy for a disease with an unpredictable out-brake prognosis cannot be justified (Holmes, 2013).

To understand the inflammation hypothesis of AD, certain physiologic assumptions need to be outlined. To begin with, cell membranes consist of lipid bilayers. The most remarkable fatty acid positioned within the phosphocholine membrane is the polyunsaturated arachidonic acid (AA; Yeagle, 1985). This integral component of the membrane triggers a cellular alert system, which acts as a kind of primordial immune system. Any disturbance of membrane integrity leads to cleavage of AA esters in the membrane by the enzyme phospholipase A2. The release of AA induces a cascade of metabolites. Cyclooxygenases and lipoxygenase oxidize this acid to yield prostaglandins, prostacyclins, thromboxanes, and leukotrienes. In higher organisms, the AA system represents the main player in inflammatory processes (Moncada and Vane, 1979).

Regarding the role of the BBB in neurodegeneration, thromboxane, which activates and attracts thrombocytes, deserves specific attention. Any lesion of a vascular membrane potentially leads to inflammation and attracts thrombocytes, which attach to the membrane *via* adhesion molecules to cover the membrane lesions (Moncada and Vane, 1979). A further task of thrombocytes is to support wound healing. Apoptosis of damaged endothelial cells is part of this process, which ends with the shedding of thrombocytes.

Like high blood sugar in the context of diabetes, APP—due to its connection to AD—is also generally perceived as fundamentally “evil.” However, from an evolutionary point of view, it is implausible that a frequently occurring protein would remain conserved in the case of exclusively carrying out a severely pathologic function. Comparably little attention has been paid to the physiological role of APP in prior investigations. In the context of inflammation of the BBB, it is remarkable that the largest amounts of APP in the whole body appear in thrombocytes (Bush et al., 1990). Interestingly, also APP plays a role in this thrombocyte adhesion process. It is cleaved at the end of this process by the enzyme β-secretase, leaving back the neurotoxic Aβ42-amyloid-fragment in the vascular cell membrane (Bush et al., 1990; Vassar et al., 1999). The functional role of APP in this context has not yet been fully clarified. It is physiological in wound healing as part of the AA system. However, it is at the same time pathophysiological in neurodegeneration. We hypothesize that the formation of Aβ42 fragments going along with cleavage of thrombocytes from an inflamed BBB is a key step in the etiology of AD.

A series of diseases of the CNS, such as multiple sclerosis (Minagar and Alexander, 2003) or viral encephalitis (Dallasta et al., 1999) is caused by inflammatory processes in the brain of different origins. In connection with the inflammatory hypotheses of AD, altered permeability of the BBB or its disruption has been proposed to allow access to inflammation in the brain. Considering inflammation of BBB tissue itself as the starting point of the disease goes beyond that; providing several new aspects and tracing back inflammation hypotheses of AD to the inflammation of the BBB. Inflamed or infected endothelial cells themselves transfer the processes from the periphery to the CNS *via* their direct contact with pericytes, glia cells, and neurons. The focus on the BBB and its inflammation provide a clear mechanistic link between APP and neurodegeneration (Heneka and O’Banion, 2007). Beyond membrane stiffness and other factors, inflammation easily adds to the concept of BBB dysfunction as a key factor to transform a cerebral metabolic syndrome into neurodegeneration (Heneka and O’Banion, 2007; Bennani-Baiti et al., 2015; Heneka et al., 2015; Gleizes et al., 2016).

### The Infection-Inflammation Interplay

Disease patterns of dementia cannot be explained by sporadically occurring, heavy infections, but suggest chronic processes in any case. Although chronic inflammation can be associated with various severe diseases with underlying factors ranging from a genetic disposition to immunological problems, there is no convincing explanation for the chronic inflammatory processes in dementia lasting for years (Dunn et al., 2005; Chitnis and Weiner, 2017). At this point, a remarkable correlation within the AA system deserves attention. This system is not only involved in the induction of inflammation in higher organisms but can also be found in microorganisms, where it is strongly involved in cell-cell interaction. If microorganisms are present in blood or tissue, they might be easily attracted by thromboxane—released from an endothelial lesion—in the same way as thrombocytes are attracted. Remarkably, they will trigger further inflammation of the cell membrane through the activation of phospholipase or their AA system. For instance, in the case of chlamydia (that has been detected in atherosclerotic plaques), their attachment to the vascular endothelium takes place *via* the action of adhesion molecules, just like in thrombocyte-attachment. Bacteria can trigger inflammatory processes, just like any other trigger of inflammation (Ramirez, 1996).

Each infection starts with the adhesion of the micro-organism to a cell membrane, usually followed by invasion. Brain infection is frequently preceded by an infection of the BBB. Chronic brain infection is one of the theories to explain the etiology of AD (Holmes, 2013), with several microorganisms proposed as candidates. There are recent reports on promising results with anti-infective treatment (Tzeng et al., 2018). However, the search for the reason of chronification in dementia remains the same, both with infection and inflammation (Dominy et al., 2019). An ongoing interplay between inflammation and infection starting from the BBB provides a good explanation for long term maintenance of inflammation (Holmes, 2013). It does not matter whether microorganisms are the immediate inflammation trigger or just opportunistic colonizers of inflamed tissue and plaques. Even a treated infection might reappear if the chronic inflammation remains because persisting quiescent microorganisms can be re-activated by prostaglandins and thromboxane to carry on the chronic process. The parallel course of inflammation and infection in a latent or chronic disease is well-known and described on an empirical basis. The mechanistic explanation linking these processes based on the AA system was therapeutically implemented for the first time with recurrent vulvovaginal candidiasis (RVVC), where in combination with a constant dose of the antifungal, the disease could be suppressed only by simultaneous treatment with a non-steroidal anti-inflammatory drug (NSAID) in a dose-dependent manner (Noe-Letschnig, 2019).

### Membrane Pore Formation and Cell Death

Within the concept of the cerebral metabolic syndrome, chronic inflammation, and/or infection of the BBB aggravate the process leading both to VD and AD. While infarcts terminate pathologic processes in VD, the situation is different for AD, where amyloid plaques appear. It is known that chronic inflammation can lead to serum amyloid A protein (SAA) induced AA amyloidosis, where repetitive phases of cell death accumulate remnants of apoptotic cells. Also, AD belongs to the class of amyloidoses, with amyloids formed from Aβ42 fragments, which are generated from their precursor protein APP *via* proteolysis (Braak et al., 1989; Bush et al., 1990; Hommet et al., 2014).

The drugs memantine and amantadine are structurally very similar, both exhibiting a ball-like shape. Nevertheless, they are used in different indications: Memantine is used in the treatment of AD (Wang and Reddy, 2017), while the primary therapeutic indication of amantadine is influenza A. Amantadine has also been used in the treatment of Morbus Parkinson (MP; Stromberg et al., 1970). Both drugs make use of a similar mechanism of pharmacologic action: They are—apart from other reported mechanisms—rather unspecific blockers of membrane channels, preventing the uncontrolled influx of protons and ions. Memantine is delaying neuronal apoptosis by interfering with excitotoxic cellular calcium influx *via* glutamatergic N-methyl-D-aspartate (NMDA) receptors (Neuhaus et al., 2011), while amantadine is blocking the matrix protein 2 of influenza 1 virus, which weakens infected cells by proton influx, thus disturbing the electrochemical potential (Bischofberger et al., 2009). The identical channel blocking mode of the pharmacologic action of these both drugs points to a potential role of APP and its Aβ42 fragments in driving damaged cells into apoptosis. The Aβ42 fragment is one of the main plaque-components in the brains of AD patients (Magalhaes et al., 2018). There has been extensive work to investigate how Aβ42 exerts its toxic function (Younkin, 1998). It is known that it tends to form oligomers. The formation of membranous pores to disturb the cell potential and pore-forming in microbial infection are known (Borlikova et al., 2013; Brown and Bevan, 2016). Because of the infection-inflammation interplay, we hypothesize that cleaved membrane sequences of both microbial origin and human APP origin can equally contribute to the apoptotic disturbance of the electrochemical potential of infected neurons.

### Chronic Infection Ending up in Amyloids

At this point, the hypothesis emerges that the physiological role of APP in thrombocytes might consist of supporting apoptosis of damaged cells *via* the formation of pores. These pore-forming domains will rest in the membrane after cleavage of APP by β-secretase. In the course of a chronic infection-inflammation process, they accumulate and are set free from disintegrating membranes during apoptosis, thus becoming infectious β-amyloid fragments, which on the one hand, may aggregate as remnants to the known amyloid plaques, or on the other hand, integrate into surrounding cell membranes. Consequently, chronic inflammation-infection of the BBB could be the prerequisite for the massive, long-lasting build-up of infectious, pore-forming β-amyloid fragments, explaining the dramatic progression of the disease. With infectious Aβ42, a new quality of disease is emerging. The inflammatory-infectious process cannot be eliminated anymore by just terminating inflammation and infection.

At this stage, genetic predisposition matters, because the tendency of Aβ42 towards protein aggregation depends on the sequence of amino acids favoring aggregation-prone β-sheet formation. Equally, the type of microbial infection might play a role. Since APP is a player in thrombocyte action in general, physiological mechanisms to prevent infectious Aβ42 formation or to remove it from the body must exist. At present, it is not known why and when a chronic inflammation, particularly in the case of Aβ42, turns into an uncontrollable process. While according to the cerebral metabolic syndrome concept, prevention and treatment of early AD is a feasible task to be solved, unfortunately, the reversion of the auto-dynamic infection by Aβ42, which is the prerequisite to stop AD at later stages, has not yet been achieved (Deyts et al., 2019).

Recently, the efflux of disease driving metabolites from the brain across the BBB has received increased attention (Poetsch et al., 2010a). Therapeutic concepts to prevent and resolve Aβ42 oligomerization are a promising approach in this direction, albeit far from the therapeutic implementation (Poetsch et al., 2010b). Removal of undesirable metabolites and deposits, including Aβ42 fragments, is a valid therapeutic concept. Amongst others, P-glycoprotein at the BBB has become a target of research related to the export of amyloid peptides (Neuhaus et al., 2010; Saidijam et al., 2018). Current concepts also consider an important role of the glymphatic system of the brain (Smith and Verkman, 2018).

## Gender Differences in Endothelial Dysfunction and Dementia

AD and other types of dementia are reported to be more frequent in women than in men. Although several limitations in prevalence and incidence studies, as well as geographical and methodological differences, need to be taken into account, there is evidence supporting the hypothesis of a greater biological risk of dementia in women including elevated tauopathy and faster brain atrophy (Buckley et al., 2019). The most striking gender-dependent biological risk factors for disadvantageous endothelial characteristics among women will be outlined in this paragraph.

Regarding the risk of dementia, microvascular defects, and certain metabolic conditions like obesity, diabetes, or high cholesterol levels seem to have a more significant pathologic impact on women than on men (Azad et al., 2007). Women show a greater risk of diabetic complications, including myocardial infarction, depression, and coronary heart disease. These complications *per se* represent some of the most evident risk-factors for AD (Kautzky-Willer et al., 2016). Further, genetic preconditions like, e.g., Apolipoprotein E ε 4 genotype status, seem to have a more pronounced (harmful) effect on hippocampal atrophy and cognitive decline in women compared to men (Fleisher et al., 2005). Thereby, the risk of conversion from physiological cognition to mild cognitive impairment, as well as from mild cognitive impairment to AD, seems to be elevated in women (Altmann et al., 2014). Besides genetic and metabolic risk factors, several hormonal studies implicate a neuroprotective role of estrogen (Petanceska et al., 2000; Rosario et al., 2011). However, inconclusive data on estrogen replacement therapy in postmenopausal women has aroused interest in other hormones of the hypothalamic-pituitary-gonadal axis that cross the BBB, e.g., gonadotropins, suggesting higher gonadotropin levels as a potential cause for AD (Webber et al., 2005). Finally, the prevalence of major depression is twice as high in women as in men (Rai et al., 2013). In this context, stress and its molecular counterpart, namely cortisol, as well as the receptor binding of corticotropin-releasing factor (CRF) has been discussed (Rosinger et al., 2020) to play a major role in the pathophysiology of depression and AD.

## Diagnosis of Dementia

### The Requirement for Early Diagnosis

The diagnosis of dementia has traditionally been clinical. Scores for mental activity have been dominant in AD diagnosis but will determine the diagnosis only in a stage of manifest disease. Despite their potential to reveal AD at early stages and to differentiate between subtypes of dementia, neuroimaging methods do not belong to the standard repertoire of routine screening procedures (Teipel et al., 2018), unless a family history of early-onset dementia is known. Over the years, also a series of clinical metabolic parameters in blood and liquor have been established (Vos et al., 2015; Wiltfang, 2015; Frölich et al., 2017). Genetic analyses aim to predict the risk of developing such a disease. Despite these diagnostic modalities, also neuroimaging techniques are available to reveal cerebral metabolic deficits, functional impairment, and protein deposits. Currently, molecular neuroimaging with positron emission tomography (PET) and ^11^C-radioligands are mainly research tools and restricted to a few medical centers in most of the countries, however, these tools provide unique opportunities in the (ultra)early diagnosis of AD to foster the investigation and implementation of causal therapeutic strategies in the pathogenesis of AD, especially regarding inflammatory processes at the BBB. The massive financial investments in the development and clinical establishment of ^18^F-radioligands for the quantification of β-amyloid plaques and tau proteins using PET are showing high confidence in this cutting-edge technology, triggering new evidence-based early diagnosis approaches and the systematic development of clinically useful prediction markers in AD.

### Positron Emission Tomography (PET) as a Diagnostic Tool in Dementia

Brain imaging methods provide a straightforward way for diagnostics in the otherwise not easily accessible brain. To reveal distinct patterns of atrophy, hypometabolism, or pathologic accumulation of β-amyloid plaques in diverse neurodegenerative disorders involving dementia, various neuroimaging modalities exist. Especially, PET plays a crucial role in the diagnosis and differentiation of relevant pathophysiologic entities. In this regard, diverse target points evolved. FDG-PET, targeting specific brain areas with pathologic glucose hypometabolism, uses fluorodeoxyglucose ([^18^F]-FDG) as a radioligand. Amyloid-PET scans need β-amyloid-specific tracers to visualize Aβ-40 and Aβ-42 deposits (Berndt et al., 2008). Regarding the imaging of inflammatory processes in AD and other pathologies linked to dementia, translocator-protein (TSPO)-tracers (Hommet et al., 2014) are used. Moreover, the efflux transporter P-glycoprotein (P-gp), limiting substrate compounds at the BBB, and maintaining the homeostasis of the brain can be examined with [^11^C]-verapamil (Hendrikse et al., 1998). P-gp is known to be frequently affected by neurodegeneration. Further approaches comprise, among others, the challenging visualization of tau protein (Villemagne et al., 2012) or targeting metabotropic glutamate receptors ([^18^F]-FPEB; Bélanger et al., 2008), the fatty acid amide hydrolase (e.g., [^11^C]-MK-3168) and acetylcholine esterases (e.g., [^11^C]-PMP; Iyo et al., 1997).

FDG-PET is known to highlight the distribution of hypometabolic brain regions in diverse types of dementia (Foster et al., 2007). Particularly [^18^F]-FDG PET is a valuable diagnostic tool to optimize the early and differential diagnosis of various pathologies comprising dementia (Mosconi et al., 2008). A meta-analysis, including 119 studies on diagnostic modalities in general, and 27 studies investigating the specific role of FDG-PET (Bloudek et al., 2011), could reveal the diagnostic superiority of FDG-PET compared to clinical diagnostic tools, CT, SPECT, and MRI. Compared to non-demented controls, a sensitivity of 90% and a specificity of 89% could be shown. Distinguishing patients with AD from patients suffering from other forms of dementia, FDG-PET reached a sensitivity of 95% and a specificity of 78% (Bloudek et al., 2011). Since glucose is both tracer and target of analysis, FDG-PET seems to be a perfect approach to develop methods for diagnosis of a cerebral metabolic syndrome and to detect early signs of metabolic impairment in the brain.

Despite the accuracy of FDG-PET concerning cerebral metabolic alterations in patients with dementia, the differentiation of AD and other entities (e.g., frontotemporal dementia) remains challenging. Due to anatomic overlaps with regards to hypometabolic brain regions in various types of dementia and β-amyloid fragments specifically characterizing AD, targeting these β-amyloid plaques is helpful to identify individuals with AD (Rabinovici et al., 2011). Therefore, various tracers can be used (e.g., [^11^C]-PiB; Klunk et al., 2004 and [^18^F]-Florbetapir; Berndt et al., 2008; Clark et al., 2011).

### Diagnosis of Neuroinflammation by PET

As described earlier, neuroinflammatory processes play a major role in multiple neurodegenerative diseases. Neuronal decline due to inflammation is triggered by the activation of microglia, inducing the production of free radicals (Klegeris and McGeer, 2000), the stimulation of T cells (Togo et al., 2002), and thereby the release of pro-inflammatory cytokines (e.g., IL-6, IL-1β, and TNF-α; Swardfager et al., 2010). In this sense, complement activation by β-amyloid (Rogers et al., 1992) and tau protein (Holmes and Diamond, 2014; Stancu et al., 2019) seem to play a crucial role. This pattern might be triggered by infections, like herpes simplex virus (Hogestyn et al., 2018), helicobacter pylori (Doulberis et al., 2018), or chlamydia pneumoniae (Lim et al., 2014), as intensely discussed in the last two decades. One way to identify these processes *via* PET is targeting the translocator protein-18 kDa (TSPO) with a large group of more than 80 suitable tracers, however, (R)-[^11^C]-PK11195 remains the most frequently used ligand in clinical practice (Damont et al., 2013). TSPO is known to be upregulated in the presence of neurodegenerative pathologies and is associated with microglial activation. Establishing and validating methods to detect inflammatory processes specifically at the microvascular endothelium of the BBB will be a challenging, but certainly rewarding task.

### Magnetic Resonance Imaging (MRI)

It is evident that imaging methods currently represent the gold standard approach in diagnosing brain diseases beyond clinical evaluation and specific liquor/blood parameters. In this context, also the potential of imaging methods beyond PET, such as MRI, is of importance. In that respect, structural MRI is a frequently applied diagnostic tool to assess structural changes like generalized brain atrophy (Fox et al., 1999) and e.g., hippocampal atrophy (Kesslak et al., 1991) as well as microvascular alterations in the diseased brain (Christov et al., 2008). Although not established in clinical practice, functional MRI (fMRI) provides an evidentially effective possibility to record network connectivity changes, detect relevant cortical hubs (Buckner et al., 2009) and uncover functional impairment both in the resting (Greicius et al., 2004; Supekar et al., 2008) as well as the task performing brain (De Marco et al., 2017). Therefore, it can be expected that future investigations might pave the way for the clinical implementation of fMRI as an elementary low-cost (compared to PET) diagnostic tool, also for early stages of cognitive impairment and AD.

### Other Diagnostic Methods

The importance of early diagnosis in the case of dementia is already known in principle, with a broad repertoire of various methods already available and constantly evolving. The need to distinguish between transient cognitive impairment and progressive forms of dementia as soon as the first symptoms occur is challenging. In the context of the cerebral metabolic syndrome and the upcoming option for a causal therapy of early dementia, it is to be expected that the scope of potential differential methods for diagnosis might become rather broad, ranging from smelling tests (Devanand et al., 2000) and pupillometry (Fotiou et al., 2000) to polymerase chain reaction PCR (Goate et al., 1991).

## Conclusion

Increased consideration of the role of brain metabolism in dementia as presented here, favors synoptic approaches in diagnosis and systemic therapy of dementia. Within the concept of the cerebral metabolic syndrome, a plethora of findings of dementia that have emerged over the last decades can be interpreted in a much broader and coherent context when compared to the target-specific approaches of conventional pharmaceutical research.

The focus on the impairment of the BBB plays a central role in the development of cerebral metabolic syndrome and its sequelae. Within this concept, the etiology of dementia is rather based on metabolic than on genetic factors. Deficiency in glucose and nutrients inside the brain weaken the neuronal metabolism and provoke disease progression. Just as in the case of the general metabolic syndrome, preventive measures are of special importance to hold back pathological processes at the beginning of the disorder. Support to the choline metabolism may further specifically inhibit apoptotic processes and help to delay the progression of dementia.

Beyond membrane stiffness induced by nutritional behavior and a harmful lifestyle (e.g., smoking), chronic inflammation and/or infections of the BBB can be considered as the main reasons for its dysfunction. Nevertheless, both treatment of AD with anti-inflammatory drugs and/or with anti-infectives could not provide a therapeutic breakthrough, hitherto. The thrombocyte protein APP links the inflammation of the BBB directly to neurodegeneration. Its physiological role in inflammatory processes is to be further clarified in general. The chronification of inflammatory processes at the BBB *via* inflammation-infection interplay promotes apoptotic processes and amyloid deposition. The understanding of this mechanism needs to be deepened. In this context, self-supported autoinfection by Aβ42-fragments of APP will play an important role. In addition to prevention and early therapy, termination of the Aβ42 induced apoptotic process is an indispensable final step to complete the option for a causal therapy of AD, even at later stages (Poksay et al., 2017).

Given the growing prevalence of dementia in the aging population, an appropriate lifestyle is a key factor in preventing the outbreak of neurodegeneration. The cerebral metabolic syndrome points towards the essential criteria to maintain mental health. Health competency and self-awareness among the general population need to be promoted to call attention towards the personal responsibility to preserve mental health.

It seems a feasible task to treat neurodegeneration effectively shortly. Early diagnosis is a key prerequisite for successful causal therapeutic intervention (Poetsch et al., 2010a). In contrast to all the stagnating efforts in conventional AD research, the definition of the cerebral metabolic syndrome will contribute to widening the view and open up the way for preventive and therapeutic concepts that can be implemented swiftly.

## Author Contributions

All authors of the manuscript were involved in the manuscript conception, revised several versions, and approved the final version. All authors contributed to the article and approved the submitted version.

## Conflict of Interest

RL received conference speaker honoraria within the last 3 years from Bruker BioSpin MR, Heel, and support from Siemens Healthcare regarding clinical research using PET/MR. CRN, MN-L, and RL are shareholders of the start-up company BM Health GmbH since Feb. 2019. MN-L was employed by the company ProFem GmbH. The remaining authors declare that the research was conducted in the absence of any commercial or financial relationships that could be construed as a potential conflict of interest.
